# Cardiorespiratory fitness, occupational aerobic workload and age: workplace measurements among blue-collar workers

**DOI:** 10.1007/s00420-020-01596-5

**Published:** 2020-11-08

**Authors:** Matthew Leigh Stevens, Patrick Crowley, Andreas Holtermann, Ole Steen Mortensen, Mette Korshøj

**Affiliations:** 1 The National Research Centre for the Work Environment, Copenhagen, Denmark; 2 Institute of Sports Science and Clinical Biomechanics, University of Southern Denmark, Odense, Denmark; 3 Section of Social Medicine, Department of Public Health, University of Copenhagen, Copenhagen, Denmark; 4 Department of Occupational and Social Medicine, Copenhagen University Hospital Holbæk, Holbaek, Denmark

**Keywords:** Heart rate reserve, Objective measurement, Physical work demands, Sustainable employment, Recovery

## Abstract

**Background:**

The knowledge, from laboratory studies dating back to the 1950s on the importance of the association between cardiorespiratory fitness and aerobic workload for workers health, is fundamental for promoting sustainable healthy employability among ageing blue-collar workers today. However, the association between cardiorespiratory fitness and aerobic workload has not yet been documented during daily work, and we do not know if it applies to the normal work of blue-collar workers in different age groups. We aim to investigate the association between cardiorespiratory fitness and aerobic workload among blue-collar workers using measurements of 24-h heart rate collected over consecutive working days.

**Methods:**

We analyzed baseline cardiorespiratory fitness, assessed using a sub-maximal cycle ergometer test, and 1–4 days of 24-h heart rate measurement from 497 blue-collar workers participating in the DPHACTO study. We investigated the association between cardiorespiratory fitness and aerobic workload defined as the average percentage of heart rate reserve (%HRR), maximum %HRR and the duration time spent at a high HRR (> 30%) during working hours. The association was assessed using multivariate linear regression models adjusted for age, sex, self-rated health, shift-work, prescription medication and occupation, as well as for different age strata.

**Results:**

Higher cardiorespiratory fitness was significantly associated with decreased mean %HRR −0.32 [95% CI −0.39 to −0.25], maximum %HRR −0.35 [95% CI −0.45 to −0.25] and time spent at  ≥ 30% HRR; −1.8% [95% CI −2.2 to −1.5%]. These associations were evident across age groups, with slightly stronger associations for workers aged 46–51 (total range 18–68).

**Conclusions:**

Higher cardiorespiratory fitness was associated with the decreased aerobic workload during normal work across all age groups and levels of work intensity. Our findings highlight the importance of cardiorespiratory fitness when considering the workload and its relevance in the promotion of healthy sustainable employment.

**Electronic Supplementary Material:**

The online version of this article (10.1007/s00420-020-01596-5) contains supplementary material, which is available to authorized users.

## Introduction

“Fit for work” is a fundamental concept in the understanding of relations between occupational physical activity (OPA) and health. Accordingly, the importance of high cardiorespiratory fitness among blue-collar workers is well established (Holtermann et al. 2010a). This is exemplified in research showing that high OPA increases the risk for cardiovascular disease mortality among workers with low and moderate cardiorespiratory fitness, but not among workers with high cardiorespiratory fitness (Holtermann et al. 2010b). Such findings show that high cardiorespiratory fitness has a protective effect among blue-collar workers with high OPA.

The underlying mechanism for the protective effect of high cardiorespiratory fitness is thought to be due to the relatively lower aerobic workload for a fit, compared to an unfit, worker doing the same manual work task. However, this knowledge is based on the findings of classical standardized laboratory studies measuring aerobic workload during graded exercise (Karvonen 1957). Thus, it remains unknown whether this basic pillar of work physiology—the protective effect of high cardiorespiratory fitness—actually applies during normal unconstrained work of blue-collar workers. Moreover, because cardiorespiratory fitness decreases with age (Hodgson and Buskirk 1977; Klabunde 2011), and because we now have an ageing workforce who face the prospect of an increasing retirement age (OECD 2017), knowledge about how the relationship between cardiorespiratory fitness and aerobic workload changes throughout working life is of great importance for ensuring sufficient workability and thus sustainable employability (Ng and Chan 2018). Verifying the classical laboratory findings in an unconstrained workplace setting is an important next step in the knowledge needed for health promotion and prevention of early retirement.

A commonly utilized method for measuring aerobic workload outside of the laboratory is to measure heart rate reserve (HRR). HRR is calculated using the maximum, minimum and working heart rate estimates (Korshøj et al. 2015) and indicates the individual heart rate at work relative to the total possible span of heart rate. It thereby provides an individual estimate of the aerobic workload (i.e. physiological response to the work performed).

Using heart rate measurements collected over 1–4 consecutive workdays during normal unconstrained work among 497 blue-collar workers we investigated the association between cardiorespiratory fitness and aerobic workload, and whether this association depends on the workers age.

## Method

Data collected from the Danish PHysical ACTivity cohort with Objective measurements (DPHACTO) was used for analysis. The DPHACTO cohort consists of blue-collar workers from 15 companies across the cleaning, manufacturing, and transport sectors (Jørgensen et al. 2019). Data collection was conducted between December 2011 and March 2013 and in accordance with the Helsinki Declaration. The study was approved by the Danish Data Protection Agency and local ethics committee (H-2-2012-011). All participants provided written informed consent prior to assessment.

### Criteria for inclusion and exclusions

Eligible workplaces were workplaces that employed blue-collar workers from the cleaning, manufacturing and transportation sectors and gave consent to allow measurements to take place during working hours. Workers from eligible workplaces were invited to participate in the baseline health check, questionnaire, and subsequent diurnal measurement (Jørgensen et al. 2019). Workers were included if they responded to the baseline questionnaire, participated in the baseline health check, and agreed to 24-h activity measurement over 1–4 consecutive days. Workers were excluded from cardiorespiratory fitness testing if they suffered from hypertension—identified as blood pressure of  ≥ 160 or  ≥ 100 mmHg for systole and diastole respectively at the health check—or self-reported angina pectoris, or declared the use of heart or lung medicine. Moreover, workers with a history of disc herniation, considerable musculoskeletal pain in the lower back area, or fever on the day of testing were excluded from cardiorespiratory fitness testing. No measurements were taken in the case of pregnancy and individuals reporting allergy to bandages or adhesives were excluded from the diurnal heart rate measurements. We only included workers who provided valid data for cardiorespiratory fitness and 24-h heart rate in our analysis.

## Data collection

### Assessment of cardiorespiratory fitness

Baseline measurements included digital questionnaires, anthropometrical measurements, and objective measurements of cardiorespiratory fitness and heart rate. Cardiorespiratory fitness was assessed using a submaximal cycle ergometer test (Åstrand and Ryhming 1954), on an Ergomedic 874 E cycle ergometer (Monark AB, Varberg, Sweden). This test measures the heart rate required to achieve a given power output (work conducted) to provide an estimate of *V*O_2max_ that takes into account both age and gender. Initial power output estimation was made using the information on age and estimated fitness. Typical output was estimated at between 60 and 90 W at a cadence of 60 revolutions per minute. Tests lasted a maximum of 10 min and were terminated once a steady-state heart rate was registered—defined as a heart rate change of less than 5 beats/min from 5 to 6 min. If a heart rate of less than 110 beats/min was registered after the first minute, power output was increased to achieve a registered heart rate at or above 60% of the estimated maximum or at least 120 beats/min. Heart rate was measured with a handheld pulse oximeter (Nellcor OxiMax N-65, United States), fixed to the fingertip. The combination of power output and heart rate were then used in the estimation of maximal oxygen uptake (Åstrand and Ryhming 1954).

### Assessment of aerobic workload

Following baseline measurements, a single Actiheart device (Actiheart, CamNTech Ltd., Cambridge, England) was used to measure 24-h heart rate, over four consecutive days. Actiheart consists of a single electrocardiography node attached at the apex of the sternum and connected to a secondary node, which is attached over any intercostal space on the left side of the ribcage. Workers were also requested to log work, leisure, and sleep periods, and periods without wearing the monitors in a diary provided after device placement. Furthermore, workers were instructed to remove any monitors causing irritation or discomfort.

For heart rate data to be considered a valid representation of aerobic workload, the Actiheart needed to be worn for at least one valid day. A valid day was defined as  ≥ 4 h of heart-rate data collection during work hours or  ≥ 75% of the individual's average work hours. We also excluded measurements with beat error  > 50%.

The calculation of heart rate reserve (%HRR) was as follows (Karvonen 1957):

1 $$ {\text{\% HRR}}\, = \,\frac{{{\text{HR}}_{{{\text{HRwork}}}} \,{ - }\,{\text{HR}}_{{{\text{HRmin}}}} }}{{{\text{HR}}_{{{\text{HRmax}}}} \,{ - }\,{\text{HR}}_{{{\text{HRmin}}}} }} \times 100{ } $$

where HR_min_ was the minimum heart rate over an average of ten beats using a moving window during the course of the whole measurement period and HR_max_ was estimated according to (Tanaka et al. 2001):

2 $$ {\text{HR}}_{{\text{max }}} = 208 - 0.7 \times {\text{Age }} $$

We chose to use HR_min_ because heart rate, like blood pressure, can be much influenced by mental stress or physical activities if measured in a clinical health setting. Since we measured the heart rate continuously during the 24 h of the day, we could therefore subtract the minimum heart rate throughout the 24 h. Thus, to attain the most precise estimation of HRR we used the lowest heart rate period over the day, which we believe provides the most valid estimate of the true sleeping heart rate of an individual.

Actiheart devices were initialized and downloaded using Actiheart software. All were set to short-term recording mode, allowing inter-beat intervals to be captured for up to 440,000 heartbeats. Once downloaded, the inter-beat intervals recorded on Actiheart were further processed and checked for errors using Acti4 software using methods described in full previously (Kristiansen et al. 2011). In brief, Acti4 software splits 24-h heart rate measurements into periods of work, leisure, and sleep time based on the information contained in participant diaries (Skotte and Kristiansen 2014). Inter-beat intervals were then resampled at a 4 Hz frequency through the implementation of a linear interpolation scheme and the calculations outlined above were completed (Eqs. 1, 2).

Inter-beat intervals corresponding to less than 36 beats/min or greater than 200 beats/min were discarded (Skotte and Kristiansen 2014). Additionally, intervals differing by more than 15% compared to their neighbouring intervals or containing an error rate greater than 50% were discarded (Skotte and Kristiansen 2014).

### Assessment of covariates and other variables

Age was determined based on the date of birth of the participant and sex was determined using the question “Are you male or female?” self-rated general health was determined by the question “How will you rate your overall health?” rated on a scale of 1 = very good, 2 = good, 3 = fairly good, 4 = poor, 5 = very poor. Participant’s use of medication was determined using the question “Have you in the last three months been taking prescription medication?” with a dichotomous ‘yes/no’ response category. This question was followed by “If yes, what kind of medication?” also using a dichotomous ‘yes/no’ response category. Occupational sector was determined according to the workplace, and occupation itself was determined by the question “What is your present main occupation?” with the response categories: 1 = blue-collar, 2 = white-collar and 3 = manager. For our analyses, these two variables (sector and occupation) were combined into a new occupation variable in the following way: blue-collar workers from each of the three occupational sectors (cleaning, manufacturing, transportation) were classified according to their sector, while white-collar workers and managers from each of the sectors were classified together as ‘administration workers’. Shift workers were identified with the question “At what time of the day do you usually work in your main occupation?” with category responses “fixed day work” and “night/varying/other”. Participants reported their workability on a 0–10 scale in response to “Please rate your present work ability?”.

### Outcomes

Three outcomes were used in this study. These were; mean %HRR at work (%HRRmean), maximum %HRR at work (%HRRmax; the maximum %HRR above which the participant has spent at least 1 min), and the proportion of time at work spent at or above 30% of HRR (≥ 30%HRR). 30%HRR was chosen as a cut-off due to the suggestions of previous literature (Wu and Wang 2002; Korshøj et al. 2015). The association between the exposure (cardiorespiratory fitness) and each of these outcomes were investigated independently using linear regression modelling.

### Statistical analyses

We chose to express the time spent above and below 30% of HRR as a proportion of an approximately 8-h workday. Therefore, this data was first transformed into isometric log-ratio coordinates in accordance with the principles of compositional data analyses (CoDA). Simply put, this method allows for the time spent above and below 30% HRR to be expressed relative to each other. As such, instead of investigating only the time spent above 30% HRR, we investigate the time spent above 30% HRR relative to the time spent below 30% HRR. In this manner, we can account for individual differences in the daily composition of HRR (i.e. the proportion a 24-h day with a HRR above or below 30% of an individual’s overall HRR).

### The association between cardiorespiratory fitness and aerobic workload

The primary analysis involved two regression models for each outcome. The first was an unadjusted model that investigated the association between cardiorespiratory fitness and each HRR outcome. The second was an adjusted model that included age, sex, self-rated health, shift-work, prescription medication and occupation as potential confounders. These potential confounders were retained in the model regardless of significance. Where significant effects of potential confounders were identified (*α* = 0.05), the interaction was assessed by adding an interaction term to the model. We used scatterplots with locally weighted polynomial regression (LOWESS) lines to visualise the relationship between cardiorespiratory fitness and each outcome. Contour plots were used to visualise the effects of identified interactions when the interacting variable (i.e. age) was continuous. We chose to include LOWESS lines as they are a widely utilized method for displaying relationships between investigated variables that is unconstrained by the assumptions used in a regression model. This same logic is used for the inclusion of the contour plots, which are developed using LOWESS principles. Linear regression models were used as they provided a satisfactory fit for the data.

### Stratification by occupation, pace of work, and age

The secondary analysis involved stratification by occupational group and age. We chose to stratify by the occupational group due to differences in activity levels between each of these groups. However, this stratification revealed that there was also a wide variation in physical activity levels within occupational groups. Therefore, we decided post-hoc to further stratify according to quartiles of steps-per-hour at work as a proxy for the pace or intensity of work, thus providing a rough approximation of work pace regardless of the occupational group. We also chose to stratify by age (using quartiles) to understand the identified interaction between cardiorespiratory fitness and age. We chose quartiles to maintain a balanced number of participants between groups and because we had no specific reason to choose any other value(s). In all cases, stratification was based upon the adjusted model (i.e. all potential confounders were included in the model except the variable used to stratify). All analyses were conducted in R (R Core Team 2018)/Studio (RStudio Team 2016) using packages: compositions (van den Boogaart et al. 2018), robcompositions (Matthias et al. 2019), car (Fox 2018), lmtest (Hothorn et al. 2018), and the tidyverse suite of packages (Wickham et al. 2019).

## Results

Of the 1087 workers included in DPHACTO, 497 provided data relevant to this analysis. For our collection of heart rate data, the median (interquartile range) number of days included for each participant was 3 (2 to 3). In line with this, the mean (standard deviation; SD) number of hours at work for each participant contributing to the analyses was 18.5 (7.8). Participants had a mean (SD) age of 44 (9.8) years and 41% were women. Most (67%) were blue-collar workers employed in the manufacturing sector and nearly all (99%) reported a general health that was ‘fairly good’ or better. The mean Cardiorespiratory fitness was 32 (8.9) and the mean %HRR at work was 30 (7.4). Full details are provided in Table 1. Information on the groups stratified by age, occupation and work pace are provided in the Online Appendix.

**Table 1:** Demographics details of workers from cleaning, manufacturing and transportation sectors in Denmark (*n *= 497)

	Mean (SD), median (IQR) or *n* (%)
Age (years)	44 (SD 9.8)
Sex (f)	203 (41%)
BMI (kg/m^2^; *n* = 496)	27 (SD 4.6)
Smoking (*n* = 489)	
Never	200 (41%)
Former	146 (30%)
Current	34 (7%)
Daily	109 (22%)
General Health (*n* = 487)	
Very poor	0 (0%)
Poor	7 (1%)
Fairly good	137 (28%)
Good	301 (62%)
Very good	42 (9%)
Cardiorespiratory fitness (mlO_2_/min/kg)	32 (SD 8.9)
Occupation (*n* = 445)	
Administration	68 (15%)
Cleaning	42 (9%)
Manufacturing	298 (67%)
Transportation	37 (8%)
Shift-work (*n* = 484)	
Fixed day work	386 (80%)
Night/varying work hours	69 (14%)
Other	29 (6%)
Work ability (*n* = 495)	9 (IQR 8 to 9)
Mean %HRR at work	30 (SD 7.4)
Maximum %HRR at work	57 (SD 10.5)
Proportion of time spent > 30%HRR at work	47% (IQR 23–70%)

## The association between cardiorespiratory fitness and aerobic workload

**Fig. 1:**
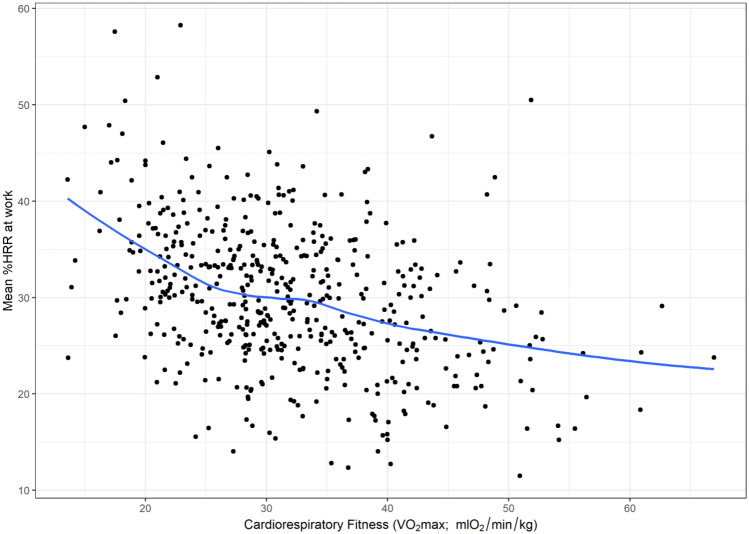
Scatterplot (and LOWESS line) showing the relationship between cardiorespiratory fitness and mean % heart rate reserve (%HRR) at work

**Fig. 2:**
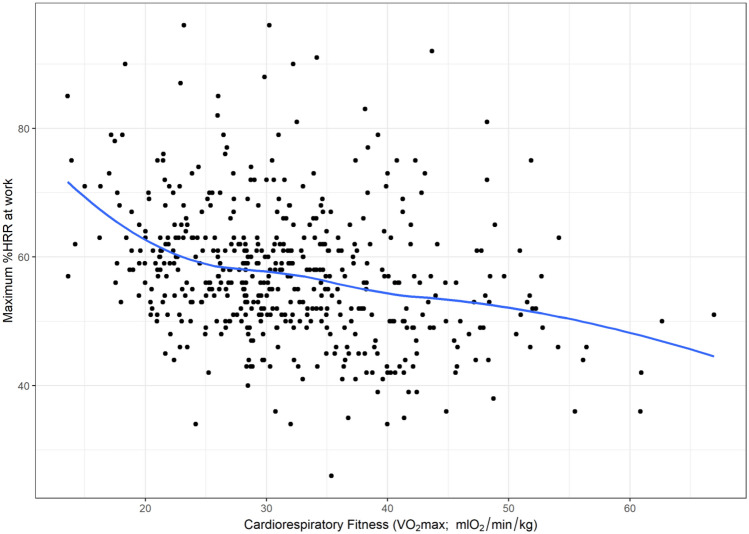
Scatterplot (and LOWESS line) showing the relationship between cardiorespiratory fitness and maximum % heart rate reserve (%HRR) at work

**Fig. 3:**
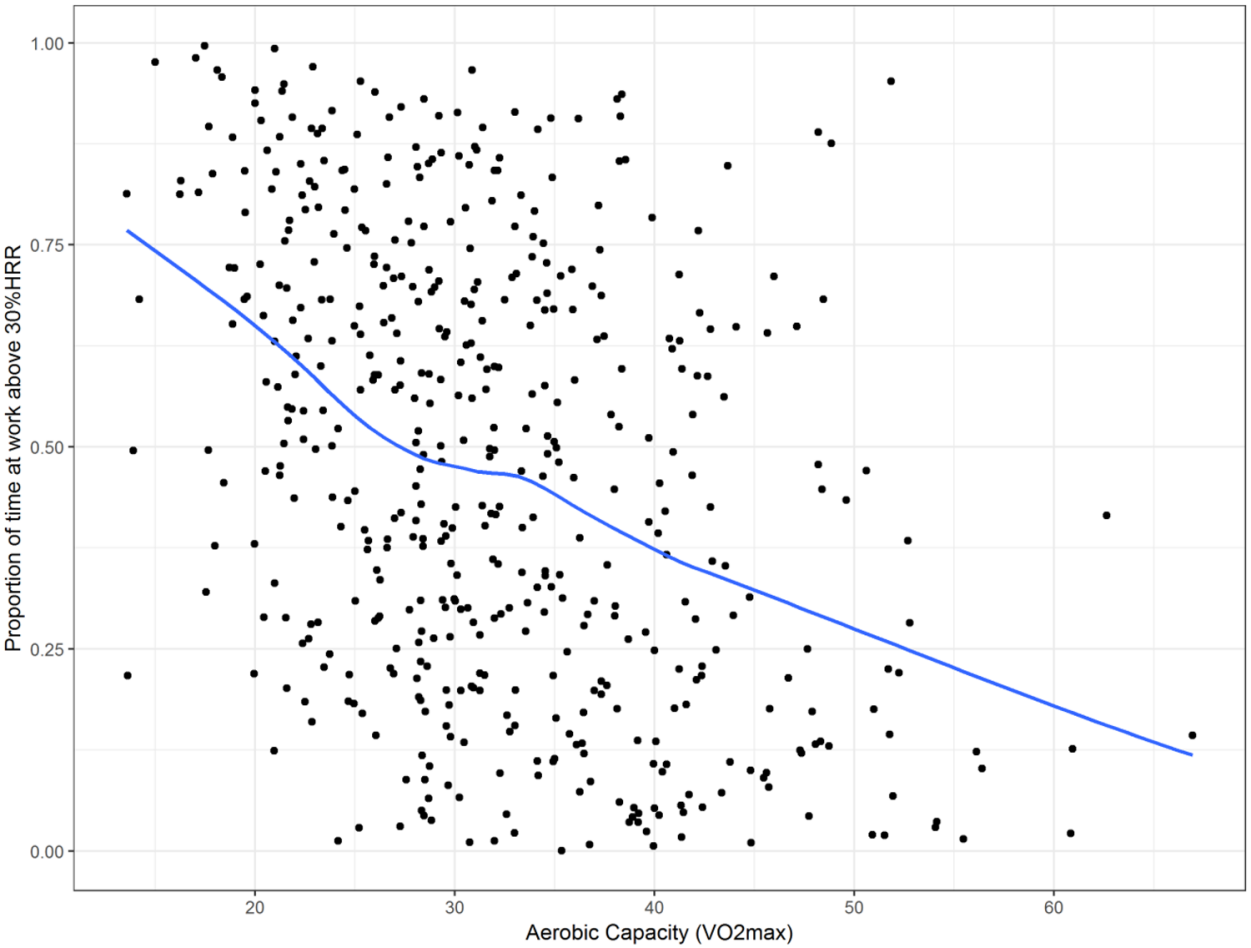
Scatterplot (and LOWESS line) showing the relationship between cardiorespiratory fitness and the proportion of time spent above 30% heart rate reserve (30%HRR) at work

**Fig. 4:**
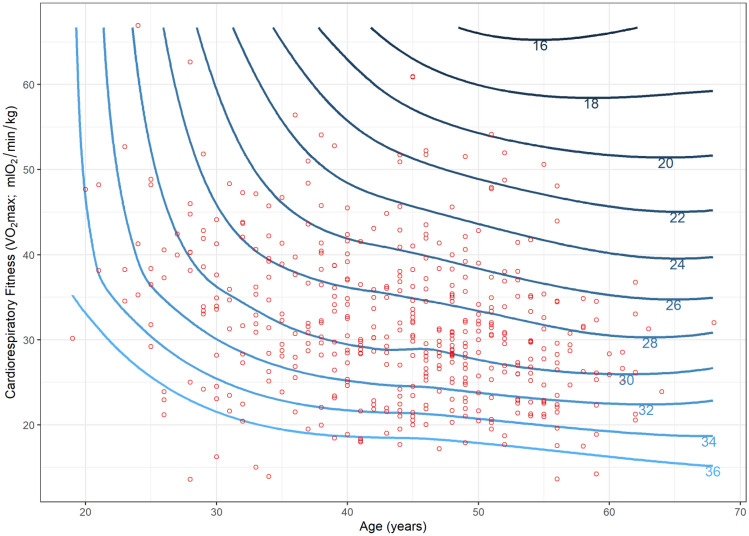
Contour plot of the interaction between cardiorespiratory fitness and age in relation to mean % heart rate reserve (%HRR_mean_) at work. Contour plots use the difference between contour lines to show the change in an outcome (%HRR_mean_, illustrated by the contour lines) in relation to two independent variables (age and cardiorespiratory fitness). For any given age, a smaller vertical distance between contour lines indicates a larger effect (greater slope) of cardiorespiratory fitness on %HRR_mean_ at work

**Fig. 5:**
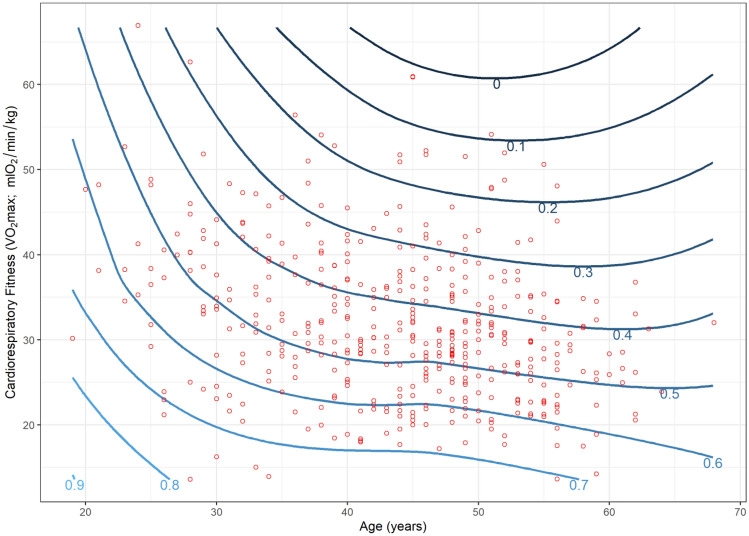
Contour plot of the interaction between cardiorespiratory fitness and age in relation to the proportion of time spent  > 30% heart rate reserve (HRR) at work. Contour plots use the difference between contour lines to show the change in an outcome (the proportion of time spent >30 %HRR at work; illustrated by the contour lines) in relation to two independent variables (age and cardiorespiratory fitness). For any given age, a smaller vertical distance between contour lines indicates a larger effect (greater slope) of cardiorespiratory fitness on the proportion of time spent >30 %HRR at work

Scatterplots (and LOWESS lines) showed a clear negative relationship between cardiorespiratory fitness and the three measures of aerobic workload (HRRmean, HRRmax and the proportion of time spent > 30%HRR) used as outcomes in our study (Figs. 1,2, 3). Regression analyses show a significant association between cardiorespiratory fitness and aerobic workload (%HRR), whereby higher cardiorespiratory fitness is associated with lower values in all three outcome measures. The estimates in the unadjusted analysis for %HRRmean, %HRRmax and  > 30% HRR were β = −0.32 [95% CI −0.39 to −0.25], β = −0.35 [−0.45 to −0.25] and Δ%*t* (change in the proportion of time spent above 30%HRR) =  −1.8% [−2.2 to −1.5%], respectively (Table 2). When we adjusted the model for age, sex, self-rated health, shift-work and prescription medication, all associations increased slightly in strength, but there were no changes in statistical significance (Table 2). Covariates identified as significant in the multivariate model were age and occupation for all outcome measures, and shift work for %HRRmean only (*p* < 0.05). Moreover, interaction effects were identified between cardiorespiratory fitness and age for both %HRRmean (*p* = 0.048) and  > 30%HRR (*p* = 0.011). Contour plots of these interactions indicate that among older age groups, increases in cardiorespiratory fitness are associated with greater reductions in aerobic workload (Figs. 4, 5).

**Table 2:** The association between cardiorespiratory fitness (*V*O_2max_) and relative aerobic load (%HRR) at work

	Estimates [95% CI]	
	Unadjusted analysis (*n* = 497)	Adjusted analysis (*n* = 439)
Mean %HRR^a^	**−0.32** [**−0.39** to **−0.25**]	**−0.35** [**−0.42** to **−0.28**]
Max %HRR ^a^	**−0.35** [**−0.45** to **−0.25**]	**−0.43** [**−0.55** to **−0.32**]
> 30%HRR^b^	**−1.8**% [**−2.2** to **−1.5%**]	**−1.9%** [**−2.3** to **−1.5%**]

Bold indicates statistically significant effects (*p* < 0.05)

*HRR* heart rate reserve, *Max %HRR* maximum % of HRR at which an individual has spent at least 1 min at or above during work, *>30%HRR* percentage of time spent at work above 30% HRR

^a^Estimates are β-coefficients

^b^Estimates are the absolute change in the proportion of time spent at work above 30%HRR

When stratified on age, all estimates remained negative and statistically significant (Table 3). When comparing across age groups, each outcome measure tended towards a u-shaped association with the strongest association between cardiorespiratory fitness and aerobic workload among the 46 to 51-year-old age group. The effect estimates in this group were *β* = −0.45 [−0.60 to −0.31], *β* = −0.45 [−0.70 to −0.20] and Δ%*t* = −2.6% [−3.4% to −1.7%] for %HRRmean, %HRRmax, and  > 30%HRR respectively. When stratified according to the occupation, the direction of effect remained the same for all analyses; however, the size of the effect did not remain statistically significant in the two smallest occupational groups, cleaning and transport (Table 4). Across all occupational groups, the effect sizes ranged from −0.37 to −0.18 for %HRRmean, −0.50 to −0.09 for %HRR_max_ and −1.0 to −2.2% for  > 30%HRR. When stratified according to the mean steps-per-hour at work, higher cardiorespiratory fitness was associated with significantly reduced aerobic workload across all outcome measures and work pace intensities (Table 5). The effect sizes ranged from −0.38 [−0.52 to −0.25] to −0.26 [−0.39 to −0.13] for %HRRmean, −0.58 [−0.79 to −0.36] to −0.33 [−0.59 to −0.07] for %HRR_max_, and −1.9% [−2.6 to −1.2%] to −1.4% [−2.2 to −0.8%] for  > 30%HRR.

**Table 3:** The association between cardiorespiratory fitness (*V*O_2max_) and relative aerobic load (%HRR) at work—stratified by occupation

Occupation group	Estimates [95% CI]			
	Administration (*n* = 68)	Cleaning (*n* = 42)	Manufacturing (*n* = 293)	Transportation (*n* = 36)
Mean %HRR^a^	**−0.37 [−0.56 to −0.18]**	−0.18 [−0.41 to 0.05]	**−0.38 [−0.47 to −0.30]**	−0.35 [−0.76 to 0.06]
Max %HRR^a^	**−0.50 [−0.84 to −0.17]**	−0.30 [−0.77 to 0.18]	**−0.48 [−0.60 to −0.36]**	−0.09 [−0.64 to 0.47]
> 30%HRR^b^	**−1.0% [−1.5 to −0.4%]**	**−1.0% [−1.8 to −0.0%]**	**−2.2% [−2.6 to −1.7%]**	−1.4% [−3.2 to 0.4%]

Bold indicates statistically significant effects (*p* < 0.05)

*HRR *heart rate reserve, *Max %HRR* maximum % of HRR at which an individual has spent at least 1 min at or above during work,*  > 30 %HRR * percentage of time spent at work above 30 %HRR

^a^Estimates are β-coefficients

^b^Estimates are the absolute change in the proportion of time spent at work above 30 %HRR

**Table 4:** The association between cardiorespiratory fitness (*V*O_2max_) and relative aerobic load (%HRR) at work—stratified by work pace (steps/hour at work)

	Estimates [95% CI]			
Work pace (steps/hour)	< 844.6 (*n* = 110)	844.6–1165.9 (*n* = 109)	1165.9–1492.7 (*n* = 110)	> 1492.7 (*n* = 110)
Mean %HRR^a^	**−0.38 [−0.52 to −0.24]**	**−0.38 [−0.52 to −0.25]**	-**0.26 [−0.39 to −0.13]**	**−0.35 [−0.49 to −0.22]**
Max %HRR^a^	**−0.33 [−0.59 to −0.07]**	**−0.58 [−0.79 to −0.36]**	**−0.41 [−0.62 to −0.20]**	**−0.46 [−0.68 to −0.24]**
> 30%HRR^b^	**−1.9% [−2.6 to −1.2%]**	**−1.8% [−2.4 to −1.2%]**	**−1.4% [−2.2 to −0.8%]**	**−1.9% [−2.5 to −1.2%]**

Bold indicates statistically significant effects (*p* < 0.05)

*HRR* heart rate reserve, *Max %HRR* maximum % of HRR at which an individual has spent at least 1 min at or above during work, * > 30 %HRR *percentage of time spent at work above 30 %HRR

^a^Estimates are β-coefficients

^b^Estimates are the absolute change in the proportion of time spent at work above 30 %HRR

**Table 5:** The association between cardiorespiratory fitness (*V*O_2max_) and relative aerobic load (%HRR) at work—stratified by age

Age group (years)	Estimates [95% CI]			
	≤ 37 (*n* = 111)	38–45 (*n* = 121)	46–51 (*n* = 112)	≥ 52 (*n* = 95)
Mean %HRR^a^	**−0.29 [−0.44 to −0.15]**	**−0.37 [−0.49 to −0.24]**	**-0.45 [−0.60 to −0.31]**	**−0.28 [−0.50 to −0.07]**
Max %HRR^a^	**−0.39 [−0.60 to −0.17]**	**−0.45 [−0.66 to −0.25]**	**−0.45 [−0.70 to −0.20]**	**−0.42 [−0.69 to −0.16]**
> 30%HRR^b^	**−1.5% [−2.1 to −0.8%]**	**−1.9% [−2.5 to −1.3%]**	**−2.6% [−3.4 to −1.7%]**	**−1.7% [−2.8% to −0.5%]**

Bold indicates statistically significant effects (*p* < 0.05)

*HRR* heart rate reserve, *Max %HRR* maximum % of HRR at which an individual has spent at least 1 min at or above during work, *>30%HRR* percentage of time spent at work above 30% HRR

^a^Estimates are β-coefficients

^b^Estimates are the absolute change in the proportion of time spent at work above 30%HRR

## Discussion

Our results show that higher cardiorespiratory fitness is associated with a lower aerobic workload during normal unconstrained work among blue-collar workers. This association was found regardless of age or the number of steps per hour (as a proxy for work intensity). Furthermore, we identified age as a significant moderator of the relationship between cardiorespiratory fitness and aerobic workload. This interaction tended towards a u-shaped relationship with the greatest effect of fitness on the aerobic workload being in the 46–51 years age group.

Our findings verify the results from laboratory studies dating back to the 1950s, demonstrating the importance of cardiorespiratory fitness for aerobic workload during standardized graded physical work (Karvonen 1957). To our knowledge, this is the first time this association has been verified using device-worn measurements gathered over consecutive workdays in a large sample of workers. The persistence of the association between cardiorespiratory fitness and aerobic workload across age and occupational strata supports this basic fundament of work physiology.

Our findings also support previous epidemiological studies reporting a strong protective effect of high cardiorespiratory fitness against health impairments (e.g. cardiovascular disease and all-cause mortality) among workers with high OPA (Holtermann et al. 2012; Harari et al. 2015; Wanner et al. 2019). Relative workload (e.g. HRR) is a strong determinant for the acute and long-term physiological adaptations and health effects from physical activity and our study supports the proposed causal chain of increased cardiorespiratory fitness lowering relative workload, and thus decreasing the risk cardiovascular disease and all-cause mortality (Krause 2010).

### Implications

The findings of our study support that cardiorespiratory fitness is of importance for the aerobic workload. Although we showed that this finding is consistent across all levels of work intensity (using the steps per hour as a proxy) (Table 5), we argue that this finding is particularly important for blue-collar workers. This is because blue-collar workers are at increased risk for health impairments and early drop-out from the working market compared with higher educated occupational groups with less physical demanding work (Sewdas et al. 2019). In other words, blue-collar workers in manual jobs (e.g. construction, manufacturing, service and eldercare) have a greater need for initiatives to reduce the aerobic workload—such as offering tailored exercise at the workplace for sustaining and improving cardiorespiratory fitness (Korshøj et al. 2016; Hallman et al. 2017; Lidegaard et al. 2018; Lund Rasmussen et al. 2018). Apart from increasing fitness, tailoring of manual work tasks to the cardiorespiratory fitness of workers may also be a solution for reducing the relative aerobic workload of individual workers—promoting balance between the capacity of workers and their tasks (which, currently, does not seem to be occurring Merkus et al. 2019; Oakman et al. 2019). Such tailoring would likely focus on equitable distribution in how work tasks are allocated (i.e., relative to capacity), but could also include other work modifications such as increased opportunity for rest breaks, shorter time at work, fewer days at work and help from fitter colleagues.

Our findings also suggest that age has an influence on the relationship between cardiorespiratory fitness and aerobic workload at work, with the strongest effect in the age span between 46 and 51 years of age. As we see no physiological explanation why the effect of cardiorespiratory fitness on HRR would decrease above this age, it is reasonable that this drop may be either due a tailoring towards less physically demanding manual work to the older workers and/or a ‘healthy worker effect’ (Mohren et al. 2010; Gommans et al. 2015; Zacher 2015). If that is the case, our findings suggest that cardiorespiratory fitness becomes more important for maintaining a healthy workload as age increases. Because cardiorespiratory fitness generally decreases with age (Hodgson and Buskirk 1977; Klabunde 2011), and because statutory retirement age in most countries is increasing (OECD 2017), the promotion of cardiorespiratory fitness as the workforce is aging is vital for achieving longer healthy working lives—particularly among blue-collar workers who generally have poor cardiorespiratory fitness (Kenny et al. 2016).

### Strengths and limitations

A major strength of the current study is the device-worn measurement of heart rate, measured over several consecutive days in unconstrained workplace settings. A potential limitation of our study is that the participants included in our study may not be representative of the general population. However, the demographics of our study are very similar to the overall DPHACTO cohort profile (Jørgensen et al. 2019). The primary difference between our study and the overall DPHACTO cohort was that participants in this study had higher self-rated health than those in the overall cohort (respectively, 71 vs. 61% of participants self-rated their general health as good or very good). This seems likely that, due to health issues, not all participants could perform the cardiorespiratory fitness test. This would have removed those workers with the worst health and may partly explain the potential healthy worker effect discussed above.

Another potential limitation is the use of a submaximal test for cardiorespiratory fitness. We chose to use a submaximal test instead of a maximal test for several reasons. First, among workers (particularly blue collar) not used to exercising, maximal heart rate and maximal oxygen consumption cannot be achieved in a single exercise test as it requires several sessions over a period to feel safe and to learn to reach peak aerobic intensity. Second, because a high proportion of the workers are not in very good health, we would have had a large number of participants ‘drop out’ from the test. These two reasons would have increased the likelihood that the maximal test would introduce bias in cardiorespiratory fitness. Furthermore, the test needed to be performed at the workplaces, which do not permit maximal testing for safety reasons. As such, we used a well-acknowledged and validated sub-maximal cycling test (Åstrand and Ryhming 1954).

Finally, as the majority of participants came from the manufacturing sector, this does somewhat limit our conclusions regarding other sectors. However, the fact that stratified analyses were performed and (while acknowledging the small sample sizes) there were no differences identified, mean that this issue is unlikely.

## Conclusion

Our study shows that higher cardiorespiratory fitness is associated with a lower aerobic workload during normal unconstrained work and that this association is present across all age groups. These findings serve to verify the fundamental laboratory studies that form the basis upon which our understanding of the importance of cardiorespiratory fitness for healthy work has been built. Our manuscript highlights the importance of cardiorespiratory fitness for reducing relative aerobic workload, particularly among blue-collar workers from ‘middle-age’ onwards. Therefore, our results support initiatives for promoting and sustaining cardiorespiratory fitness and promoting a balance between work tasks and the cardiorespiratory fitness of workers.

## Electronic supplementary material

Below is the link to the electronic supplementary material.

Supplementary file1 (PDF 309 kb)
