# Supplementary material for: Cardiorespiratory fitness, occupational aerobic workload and age: workplace measurements among blue-collar workers
Source: Int Arch Occup Environ Health. 2020 Nov 8;94(3):503–13. doi: 10.1007/s00420-020-01596-5 (PMC8032632; doi:10.1007/s00420-020-01596-5)
Supplement: Supplementary file 1 — Supplementary file1 (PDF 309 kb) [file 420_2020_1596_MOESM1_ESM.pdf]

Cardiorespiratory fitness, occupational aerobic workload and age: workplace measurements among blue-collar workers

Online Appendix

Table A1. Cardiorespiratory Fitness and Aerobic Workload of Danish workers stratified by Age, Occupation and Work Pace

	Cardiorespiratory Fitness (mlO_2/min/kg) [mean (SD)]	Mean %HRR at work [mean (SD)]	Maximum %HRR at work [mean (SD)]	Proportion of time spent at work above 30%HRR [median (IQR)]
Age				
≤37 (n = 111)	35 (9.9)	31 (7.5)	59 (10.7)	50% (30% to 71%)
38 to 45 (n = 121)	32 (8.9)	29 (7.2)	56 (10.5)	39% (22% to 65%)
46 to 51 (n = 112)	31 (7.8)	20 (7.1)	57 (10.6)	46% (20% to 71%)
≥52 (n = 95)	29 (7.5)	29 (7.7)	56 (9.4)	40% (22% to 68%)
Occupation				
Administration (n = 68)	33 (8.5)	26 (7.4)	56 (11.4)	24% (11% to 45%)
Cleaning (n = 42)	31 (9.3)	35 (6.5)	61 (12.2)	70% (55% to 83%)
Manufacturing (n = 293)	32 (9.1)	30 (6.7)	56 (9.5)	46% (23% to 68%)
Transportation (n = 36)	32 (8.2)	32 (9.2)	63 (10.2)	53% (36% to 73%)
Work pace (steps/hour)				
<844.6 (n = 110)	32 (8.8)	25 (6.9)	54 (10.9)	25% (9% to 44%)
844.6 to 1165.9 (n = 109)	32 (7.8)	28 (6.0)	55 (9.2)	34% (21% to 52%)
1165.9 to 1492.7 (n = 110)	32 (9.4)	31 (6.3)	58 (9.8)	54% (30% to 71%)
>1492.7 (n = 110)	32 (9.6)	34 (7.1)	60 (10.4)	68% (51% to 85%)

HRR Heart rate reserve; SD Standard deviation; IQR Interquartile range
